# Current insights into the hepatic microenvironment and advances in immunotherapy for hepatocellular carcinoma

**DOI:** 10.3389/fimmu.2023.1188277

**Published:** 2023-05-18

**Authors:** Ming Zhao, Hui Huang, Feng He, Xiangsheng Fu

**Affiliations:** Department of Gastroenterology, Clinical Medical College and the First Affiliated Hospital of Chengdu Medical College, Chengdu, Sichuan, China

**Keywords:** hepatocellular carcinoma, tumor microenvironment, cancer immunotherapy, immunocheck point inhibitors, adoptive cell therapy

## Abstract

Hepatocellular carcinoma (HCC) is the most common type of primary liver cancer and shows high global incidence and mortality rates. The liver is an immune-tolerated organ with a specific immune microenvironment that causes traditional therapeutic approaches to HCC, such as chemotherapy, radiotherapy, and molecular targeted therapy, to have limited efficacy. The dramatic advances in immuno-oncology in the past few decades have modified the paradigm of cancer therapy, ushering in the era of immunotherapy. Currently, despite the rapid integration of cancer immunotherapy into clinical practice, some patients still show no response to treatment. Therefore, a rational approach is to target the tumor microenvironment when developing the next generation of immunotherapy. This review aims to provide insights into the hepatic immune microenvironment in HCC and summarize the mechanisms of action and clinical usage of immunotherapeutic options for HCC, including immune checkpoint blockade, adoptive therapy, cytokine therapy, vaccine therapy, and oncolytic virus-based therapy.

## 1 Introduction

Hepatocellular carcinoma (HCC), the major type of hepatic malignancy, is the third leading cause of cancer-related death worldwide (1). Surgery is the best choice of intervention for early-stage HCC. However, most patients are not able to undergo surgery at the time of diagnosis due to the advanced disease stage. Other strategies, including chemotherapy, radiotherapy, molecular targeted medications, radiofrequency ablation (RFA), and transarterial chemoembolization (TACE), have limited efficacy against advanced HCC (2).

In the past decade, dramatic advances in immunology have modified the paradigm of cancer therapy, ushering in the era of immunotherapy. In cancer immunotherapy, the immune system is activated and attacks malignant cells through natural mechanisms, therefore avoiding damage to normal tissues, which is one of the most serious side effects of chemotherapy and radiotherapy. This attractive feature has supported the rapid application of immunotherapy in clinical practice. Currently, the most prevalent cancer immunotherapies include checkpoint inhibitors, engineered T cells, lymphocyte-promoting cytokines, and cancer vaccines. Immune checkpoint blockade (ICB) by monoclonal antibodies targeting cytotoxic T-lymphocyte associated protein 4 (CTLA-4) and programmed cell death 1 (PD-1) has been approved for several types of tumors and substantially benefited a subset of patients. However, most patients still show resistance to ICB (3). Therefore, targeting the tumor microenvironment is a rational approach for the development of the next generation of immunotherapy. This review will summarize the components of the immune microenvironment in HCC and the development of immunotherapy.

## 2 The immune ecosystem of the normal liver

The liver is an organ that is nourished by a dual blood supply from both the hepatic artery and the portal vein. Blood converges in the hepatic sinusoids, which slow the blood flow and allow interactions between immune cells and antigens from the gut microbiota and circulation (4). The liver has a strong ability to remove deleterious compounds because of its abundance of innate and adaptive immune cells, including resident macrophages (Kupffer cells [KCs]), natural killer (NK) cells, and lymphocytes, with a higher CD8+/CD4+ T cells than in the periphery (4, 5). Accordingly, the liver is an immune-modulating organ with a controlled T-cell response that is maintained by resident cells, including KCs, hepatic stellate cells (HSCs), dendritic cells (DCs), and regulatory T cells (Tregs) (6). The intrinsic immune tolerogenicity prevents the liver from mounting a hyperactive response to deleterious stimuli. On the other hand, it impedes immune surveillance and facilitates oncogenesis and progression (7).

## 3 The hepatic immune microenvironment in HCC

Up to 80% of HCC cases arise from chronic inflammation, which is driven by immune cell infiltration and resident cells, such as KCs, HSCs, and liver sinusoidal cells (8). Chronic inflammation creates an oxidative microenvironment and induces DNA damage and genetic alterations, favoring neoplasia initiation and progression (8).

**Figure 1:**
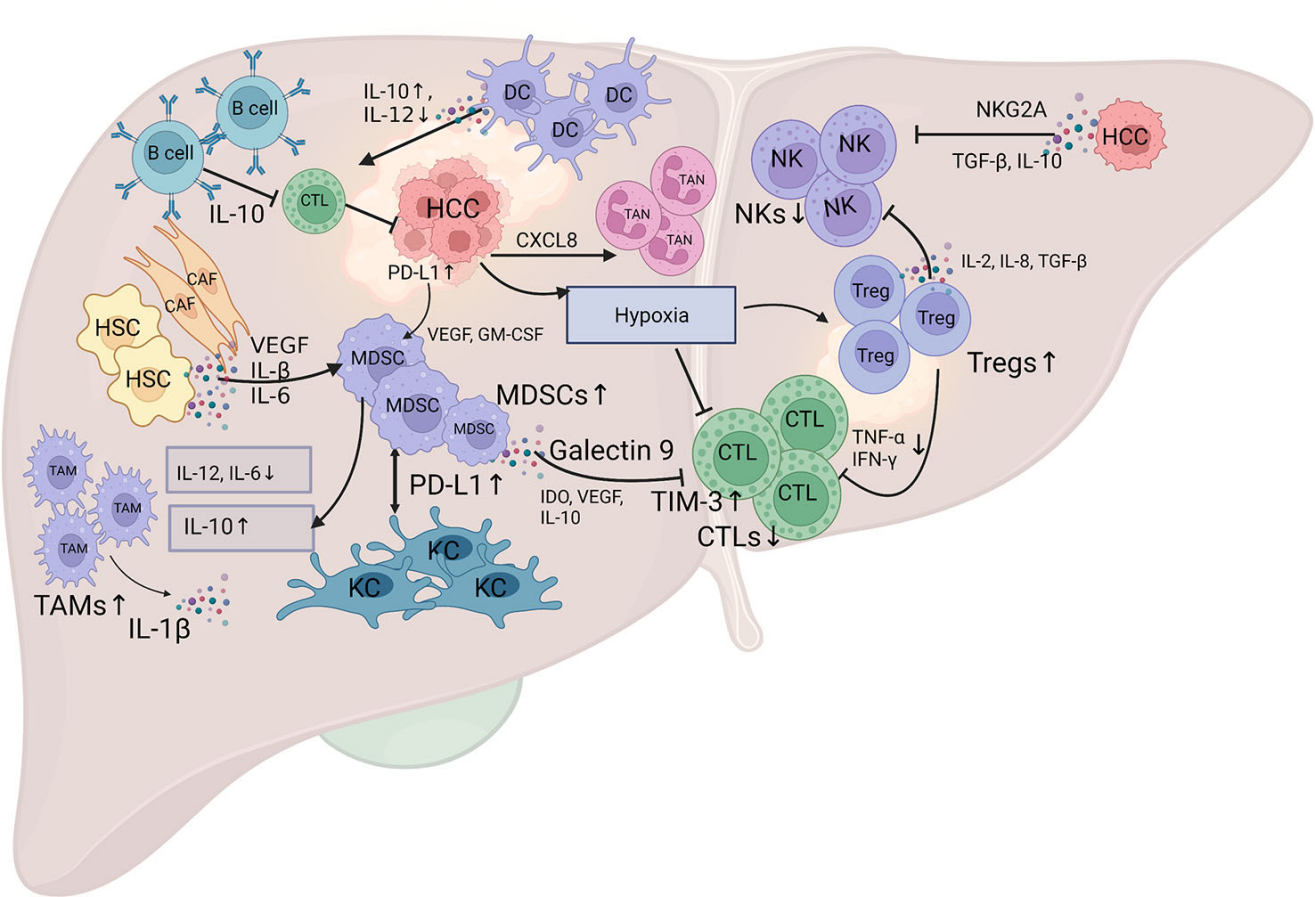
Schematic diagram of the landscape of the immune microenvironment in HCC. The infiltration of various subpopulations of immune cells, regulatory cytokines, and certain inhibitory signals mediate the specific immune response in HCC. The HCC tumor cells produce various factors, such as IDO, VEGF, IL-10, and hypoxia, to suppress the tumoricidal ability of CTL, and TGF-β, IL-10 and inhibitory receptors NKG2A, to suppress the tumoricidal ability of NK cells. Additionally, the HCC tumor cells secrete CXC chemokines, especially CXCL8, to attract TANs into the tumor stroma. The activation of PD-1 signaling in B cells promotes the production of IL-10, which inhibits the anti-tumor immunity of effector T cells. The crosstalk between MDSCs and TAMs results in a decreased secretion of IL-6 and IL-12, and an increased secretion of IL-10, which impair the cytotoxicity of CTL and NK cells. The aggregation of MDSCs in the tumor stroma is mediated by various cytokines, such as VEGF, IL-β, and IL-6 that produced by CAFs and HSCs, and VEGF and GM-CSF that produced by HCC tumor cells. MDSCs interact with KCs to induce PD-L1 expression on KCs, which interact with PD-1 on T cells to mediate immune evasion. Galectin-9 expression on MDSCs can bind to TIM-3 on T cells to induce T cell apoptosis. Tregs can inhibit CTL activation that mediated by reduced production of TNF-α and IFN-γ, and weaken the anti-tumor ability of NK cells through the production of IL-2, IL-8, and TGF-β. DCs can mediate IL-10 production and IL-12 reduction to suppress the antitumor response of CTL. CTL, cytotoxic T lymphocyte; NK, natural killer cells; TAM, tumor-associated macrophage; MDSC, myeloid-derived suppressor cell; TAN, tumor-associated neutrophil; CAF, cancer-associated fibroblast; HSC, hepatic stellate cell; KC, Kupffer cell; DC, dendritic cell; IDO, indoleamine 2,3-dioxygenase; VEGF, vascular endothelial growth factor; IL-10, interleukin-10; NKG2A, inhibitory receptors natural killer group 2A; TGF-β, transforming growth factor β; GM-CSF, granulocyte-macrophage colony-stimulating factor; ICI, immune checkpoint inhibitor. The figure was drawn at BioRender.com.

The cellular components in the immune microenvironment in HCC include several types of cells, such as tumor-infiltrating lymphocytes (TILs), tumor-associated macrophages (TAMs), dendritic cells (DCs), tumor-associated neutrophils (TANs), myeloid-derived suppressor cells (MDSCs), mast cells (MCs), HSCs, and cancer-associated fibroblasts (CAFs), as well as noncellular components, such as cytokines and the extracellular matrix (**Figure 1**).

### 3.1 Tumor-infiltrating lymphocytes

#### 3.1.1 T cells

TILs are a heterogeneous cell population comprising T cells, NK cells, and B cells. HCC patients with T-cell accumulation after surgery experience lower recurrence and longer survival (34). The accumulation of CD8+ T cells, the major cytotoxic T lymphocytes (CTLs) in the liver, is associated with reduced tumor progression and improved survival of HCC patients (35). However, their tumor-killing ability is counteracted by various factors, such as indoleamine 2,3-dioxygenase (IDO), vascular endothelial growth factor (VEGF), interleukin 10 (IL-10), hypoxia, and CD4+ T-cell deprivation (35). CD4+ T cells secrete IFN-γ and TNF-α to remove senescent hepatocytes in a CXCR6-dependent manner, thus controlling hepatocarcinogenesis (36). Accordingly, the ROS-mediated apoptosis of CD4+ T cells promotes HCC in patients with NAFLD (37). Conversely, regulatory T cells (Tregs), a subtype of CD4+ T cells characterized by the expression of CD25 and forkhead box P3 (FoxP3), are capable of suppressing the tumor immune response and are associated with the metastasis and recurrence of HCC (38). Transforming growth factor β (TGF-β) induces HCC progression by promoting Treg polarization and the consequent repression of cytotoxic CD8+ T cells (39). This phenotype was reversed by the specific TGF-β inhibitor SM-16, which induced attenuated Treg infiltration and HCC regression (39). The CTL/Treg ratio represents the immune ecosystem. A high level of CTLs might overcome Treg-mediated immune suppression, thus suppressing HCC tumor growth (40).

#### 3.1.2 NK cells

NK cells are innate lymphocytes that mediate cancer immune surveillance and eradicate tumor cells without prior sensitization (41). They account for 30%-50% of hepatic lymphocytes and play an important role in preventing fibrosis and resisting cancer and viral infections through strong cytotoxicity and the production of IFN-γ (42). With the progression of HCC, the tumor killing activity of NK cells decreases, with the characteristics of reduced expression of the NK cytotoxic factors granzyme and perforin, as well as reduced secretion of the tumor killing-related cytokines TNF-α and IFN-γ (43). The infiltration of activated NK cells with high natural killer group 2 member D (NKG2D) expression correlates with prolonged survival of patients with cholangiocarcinoma (44). In contrast, the accumulation of immature and inactivated NK cells with poor catalytic activity is associated with HCC progression (45). The catalytic activity of NK cells is counteracted by several factors, such as the inhibitory receptor NKG2A and the immunosuppressive cytokines TGF-β and IL-10 (46, 47). Specifically, a subgroup of NK cells expressing CD49a was discovered to reside in HCC tissues, with high expression of the inhibitory receptors PD1 and CD96, and was correlated with a poor prognosis of HCC (48).

#### 3.1.3 B cells

B cells directly present tumor-associated antigens to CD4+ T cells and CD8+ T cells, produce antibodies to promote the uptake of tumor antigens by TAMs and DCs, and secrete cytokines to promote antitumor immunity or directly kill tumor cells. In HCC, IgG+ memory B cells that accumulate at the edge of the invading tumor generate granzyme B, TRAIL, and IFN-γ, express surface markers of APCs, and cooperate with CD8+ T cells, resulting in a good prognosis (49). B-cell infiltration is positively correlated with survival and the response to immunotherapy (50). However, in patients with advanced HCC, activation of PD-1 signaling in B cells promotes IL-10 production and inhibits effector T-cell-mediated antitumor immunity, leading to early recurrence after tumor resection (51).

### 3.2 Dendritic cells

DCs are the major type of antigen-presenting cells that capture antigens, present them to T cells, and activate the adaptive immune response. In the healthy liver, most DCs are immature and activate effector T cells by inducing the production of antigen-specific CD8+ Tregs (52). This response might be attributed to the low level of IL-12 and high level of L-10 in the hepatic microenvironment (53). The liver contains two major groups of DCs, plasma cell-like DCs (pDCs) and conventional DCs (cDCs), which are further divided into cDC1s and cDC2s (54). cDC1s are essential for antitumor immune responses, and their presence in the tumor microenvironment is associated with a better prognosis (55), whereas cDC2s act as effective stimulators of nascent T helper (Th) cells (56). In contrast, immature pDCs in the tumor microenvironment are mainly associated with a poor prognosis for some types of human cancers (57). Compared to healthy controls, both circulating pDCs and cDCs showed a lower quantity and expression of human leukocyte antigen-DR (HLA-DR) and molecules CD80 and CD86 in the peripheral blood of HCC patients (58). Intratumorally infiltrated pDCs predict a poor prognosis among patients with primary HCC who have undergone resection, which might be caused by the induction of Treg cell and IL-17 production (59).

### 3.3 Tumor-associated macrophages

TAMs in HCC are derived from resident KCs in the liver and CD14+/CD16+ monocytes in the peripheral blood. They infiltrate into the tumor following stimulation with cytokines and chemokines, such as M-CSF, VEGF and CCL chemokines, secreted by tumor cells or mesenchymal cells (60). KCs are the major population of liver-resident macrophages with high expression of PD-L1 and low expression of CD80 and CD86, and are crucial for maintaining the immunosuppressive hepatic microenvironment (61). Antigen presentation by KCs induces the arrest of CD4+ T cells, expansion of antigen-specific Tregs and tolerogenic immunity. However, KCs lose tolerogenic markers in the injured liver. The deletion of KCs reduces hepatic tolerance to particulate antigens (61). Myeloid-derived monocytes can be attracted to infiltrate HCC, differentiate into TAMs, and acquire a phenotype similar to that of KCs. Monocyte-derived macrophages help CD4+ T cells remove senescent premalignant hepatocytes, thus inhibiting hepatocarcinogenesis (62). Conversely, senescent hepatocytes secrete CCL2 to attract CCR2+ monocytes into the liver, differentiate into macrophages and remove senescent premalignant hepatocytes (63). CD68 is a widely used marker to identify TAMs; however, it is not specific to distinguish liver-resident KCs and monocyte-derived macrophages. A recent study identified that Clecf4 and Tim4 are specifically expressed on the surface of KCs instead of monocyte-derived macrophages in HCC and thus might be used as markers for the TAM origin (64).

Similar to most cancers, TAMs in HCC are trained by specific tumor microenvironments and polarized to the M1 type and M2 type (65). M1 polarization is attributed to typical Th1 cytokines, such as interferon-γ (IFN-γ), and microbial components, such as lipopolysaccharide (LPS). M1 macrophages are classic active macrophages associated with pathogen clearance, pro-inflammatory cytokine liberation and matrix component lysis (66). M1 macrophages suppress tumor growth by directly killing tumor cells or presenting antigens to T cells. They efficiently produce pro-inflammatory cytokines, such as IL-1β, IL-6, and TNF-α, and are characterized by high expression of IL-12 and low expression of IL-10 (65). M1 macrophages secrete cytokines, such as CXCL9 and CXCL10, to attract Th1 cells, which in turn promote polarization to M1 macrophages (67). On the other hand, M2 polarization is induced by IL-4 and IL-13 (68) and is associated with parasite engulfment, anti-inflammatory cytokine release, matrix deposition and liver injury repair (66). M2 macrophages are characterized by low expression of IL-12 and high expression of IL-10 (65). Additionally, tumor-derived cytokines, such as CCL2 and CSF1, attract peripheral blood-derived macrophages to tumors and induce their differentiation to the M2 type (69). M2 macrophages are alternatively activated macrophages that secrete large amounts of anti-inflammatory cytokines, such as IL-8, IL-10, and TGF-β, to promote tumor vasculature development, CD8+ T-cell apoptosis, and the Th1 immune response, thus favoring tumor growth and metastasis (68). Notably, macrophages in peritumoral liver tissue also promote HCC progression and predict a poor prognosis (70).

### 3.4 Tumor-associated neutrophils

TANs are tumor-infiltrating neutrophils that directly influence tumor development and progression. Similar to TAMs, TANs are classified into the N1 and N2 subtypes, which have antitumor and protumor roles, respectively (71). TGF-β is the key molecule driving polarization to the N2 type with an immunosuppressive response, while TGF-β inhibition promotes N1-type polarization characterized by enhanced cytotoxicity and inflammation (71, 72). In HCC patients, tumor cells secrete CXC chemokines, especially CXCL8, to attract neutrophil accumulation in the peritumor stroma (73). CXCL5 overexpression was also reported to be correlated with neutrophil infiltration in HCC tumors and predicted a poor prognosis (74). TANs may enhance the stemness of HCC cells through the miR-301b-3p/LSAMP/CYLD axis. Conversely, stem-like HCC cells secrete CXCL5 to facilitate TAN infiltration into the tumor. The positive feedback loop between TANs and HCC stem-like cells promotes HCC progression and metastasis (75). TANs also recruit TAMs and Tregs, thus creating an immunosuppressive microenvironment to facilitate HCC carcinogenesis (76). Moreover, TANs produce an increased number of neutrophil extracellular traps (NETs) to provoke an inflammatory response and sequester circulating cancer cells (CSCs) to promote HCC metastasis (77, 78).

### 3.5 Myeloid-derived suppressor cells

MDSCs represent a heterogeneous population of immature myeloid cells that are mainly located in the bone marrow, spleen, peripheral blood, and tumors (79). Normally, myeloid cells differentiate into mature granulocytes, macrophages, or DCs to clear pathogens. However, myeloid cells cannot differentiate and become immature under conditions of inflammation or cancer. They are classified into M-MDSCs and PMN-MDSCs based on their phenotype and function (79). MDSCs create an immunosuppressive microenvironment through various mechanisms, including Treg differentiation, DC inhibition, macrophage M2 polarization, and oxidative stress (80–82). Compared with healthy volunteers or hepatitis patients, peripheral blood from HCC patients contained more MDSCs, which were subsequently revealed to indicate a poor prognosis (83). Additionally, the number of MDSCs increases with HCC progression, and these cells are closely associated with tumor size, tumor node metastasis and tumor multiplicity (84). MDSCs were reported to reduce the antigen presentation activity of KCs. In a coculture system containing MDSCs and KCs, KCs showed increased expression of CD86, CD274 and MHCII, increased secretion of IL-1β and IL-10, and reduced secretion of CCL2 and IL-18 (85). MDSC differentiation and infiltration in HCC are induced by activated hepatic stellate cells (HSCs) through the COX2-PGE2-EP4 pathway (86). HCC cells produce cytokines, such as VEGF, GM-CSF, and IL-1β, to attract MDSCs, and treatments targeting MDSCs might increase the sensitivity of immune checkpoint inhibitors (ICIs) promoting the antitumor response (87). MDSCs also reduce NK cell activity through a direct interaction to reduce NKp30 receptor expression in NK cells, which is independent of inducible nitric oxide synthase (iNOS) or arginase expression in MDSCs (88).

### 3.6 Mast cells

MCs are innate tissue-resident immune cells in the bone marrow lineage that were previously presumed to be central to the development of allergy and inflammation (89). However, MCs have recently been reported to affect tumor cells and the surrounding tumor-associated stroma, thereby altering tumor cell behavior and the tumor microenvironment (90). Previous studies have shown that total MC release, including effluent granules and histamine, reduces the viability and proliferation of HuH-6 cells (91). In addition, MCs actively participate in tumor cell clearance and tumor rejection through the release of IL-1, IL-4, IL-6, and TNF-α. Conversely, MCs release mediators, such as IL-8, FGF-2, VEGF, NGF, and PDGF, to favor tumorigenesis and angiogenesis (92). Based on their key role in orchestrating the cancer stroma, MCs have been recognized as a promising target for cancer immunotherapy.

### 3.7 Hepatic stellate cells

HSCs account for 13% of all liver cells and are located in the Disse space between hepatocytes and endothelial cells, where substance exchange between hepatocytes and blood occurs (93). HSCs are mesenchymal cells with the characteristics of adipocytes, fibroblasts, and myocytes; thus, they function to maintain vitamin A homeostasis, induce hepatofibrosis, and modulate the hepatic bloodstream (94). Normally, HSCs remain quiet to maintain vitamin A homeostasis, with low activity to synthesize the extracellular matrix (ECM) (94). Once stimulated with liver-damaging factors, such as hepatic viruses and toxic chemicals, damaged hepatocytes secrete a number of cytokines, such as TGF-β, PDGF, and TNF, to induce lipid loss and morphological changes and are transdifferentiated into myofibroblasts (MFBs) that synthesize large amounts of ECM, leading to hepatofibrosis (94). TGF-β, which is derived from various types of cells, including macrophages and even HSCs themselves, is the key stimulator of HSC activation and MFBs (95). Activated HSCs produce angiogenic growth factors, stimulating angiogenesis and establishing a new vascular system in the tumor (96). Additionally, activated HSCs recruit MDSCs and Tregs and reduce the activation of CD8+ T cells (97). HSC-mediated inflammation/MFBs are well recognized as risk factors for HCC.

### 3.8 Cancer-associated fibroblasts

CAFs are detected in almost all types of solid tumors. CAFs in tumors produce a wide variety of factors to create an immunosuppressive microenvironment that favors tumor development (98, 99). A high level of CAF-specific biomarkers, such as α-smooth muscle cell (α-SMC) actin and fibroblast-activating protein (FAP), predicts a poor prognosis for HCC patients (100). CAFs have been reported to promote cancer progression and metastasis by producing cytokines (101). Activated HCC cells produce more CCL26 to promote higher levels of CAF infiltration that facilitate HCC progression (102). In turn, CAFs induce the epithelial-mesenchymal transition (EMT) process in HCC cells, thus promoting metastasis (103). Additionally, CAFs create an immunosuppressive microenvironment by inducing MDSCs and TANs, thus facilitating immune evasion (104, 105).

## 4 Immunotherapy in HCC

### 4.1 Immune checkpoint blockade

**Figure 2:**
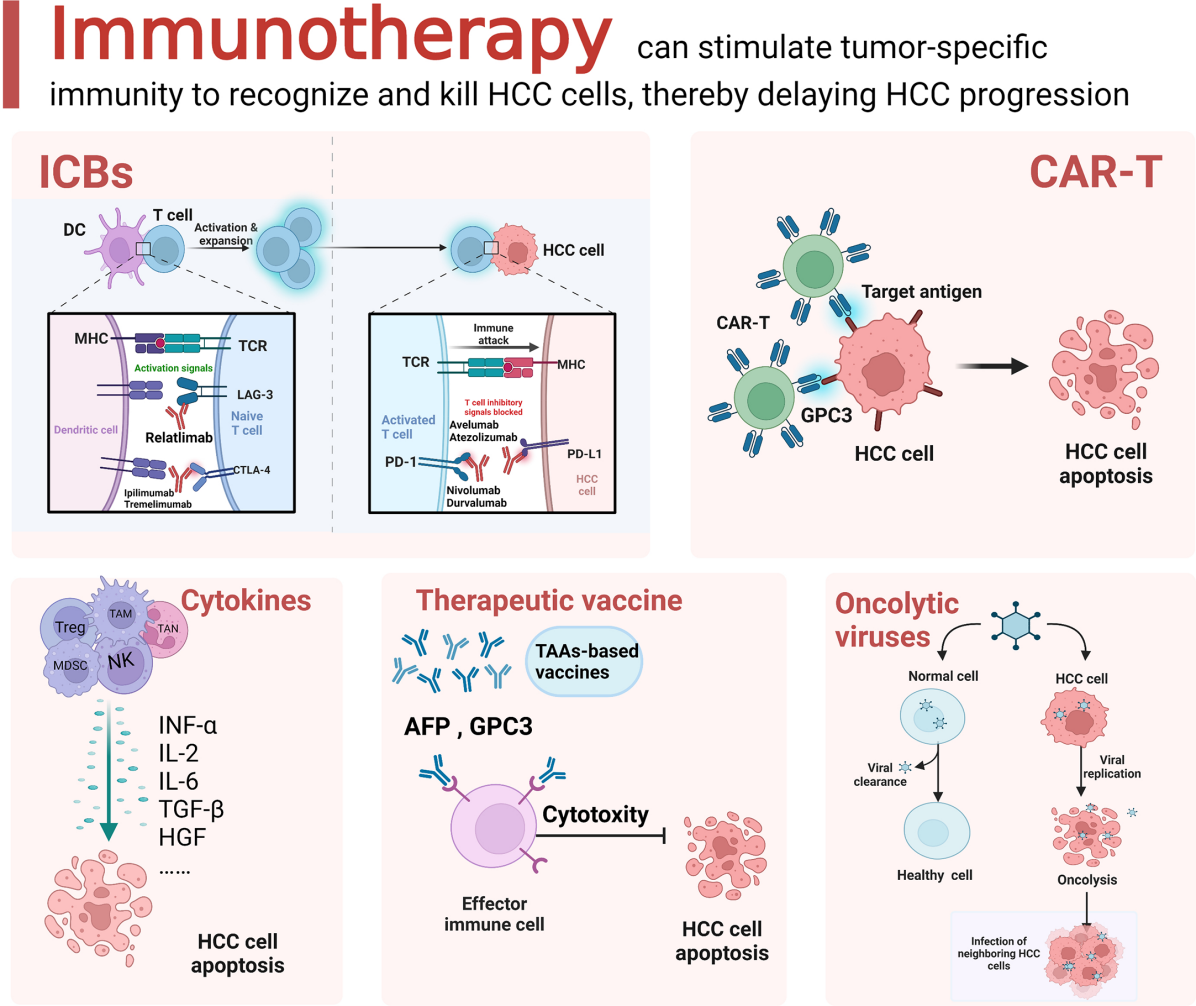
Schematic diagram of the current immunotherapeutic options for HCC. Immunotherapeutic approaches for HCC mainly include immune checkpoint blockade (ICB), adoptive cell therapy (ACT), cytokine based immune regimens, therapeutic vaccine and oncolytic virus. The figure was drawn at BioRender.com.

T-cell activation is a critical event in tumor immune therapy. Upon activation, negative costimulatory molecules, also called immune checkpoints, are expressed on the T-cell surface. The binding of these molecules to their ligands mediates coinhibitory signaling, resulting in the suppression of the proliferation and differentiation of T cells, thus promoting immune evasion (106). ICIs block ligand−receptor interactions, thus reactivating exhausted T cells to enhance the antitumor immune response (**Figure 2**).

#### 4.1.1 Monotherapy with immune checkpoint inhibitors

##### 4.1.1.1 Antibodies targeting CTLA-4

CTLA-4 is expressed primarily by Tregs and activated T cells and transmits inhibitory signals to effector T cells upon interacting with CD80/86 molecules expressed on antigen-presenting cell membranes (107). CTLA-4 outperforms CD28 in binding to B7 family costimulatory receptors (CD80/CD86) because of its 10-fold higher affinity (108), thus blocking B7/CD28-mediated costimulatory signaling (109). Tremelimumab and ipilimumab are CTLA-4 inhibitors that have shown anticancer effects on HCC patients. Tremelimumab has been shown to block signaling from the costimulatory receptor (CD80/CD86), preventing T-cell deactivation and subsequently avoiding the induction of tumor immune escape (110). In a phase II multicenter clinical trial (NCT01008358), patients with advanced HCC who were administered tremelimumab showed a disease control rate (DCR) of 76.4%, partial response (PR) rate of 17.6%, and time to progression (TTP) of 6.48 months, with good tolerance (13). Moreover, this treatment was also able to reduce the HCV load in the patient’s body (13). The most common adverse event of tremelimumab was pruritic rash, which was alleviated by antihistamine medication in most cases. These adverse events reached grade 3 or higher in 45% of cases but were unrelated to a decline in liver function (13).

##### 4.1.1.2 Antibodies targeting PD-1/PD-L1

PD-1 is the most important immune checkpoint molecule that belongs to the CD28 family. PD-L1 is the major ligand of PD-1 and is mainly expressed on the surface of tumor cells and DCs (111). The interaction between PD-1 and PD-L1 suppresses T-cell activity, induces apoptosis, and promotes Treg differentiation, thus creating an immunosuppressive microenvironment and leading to immune evasion (112). A higher level of the PD-L1 protein is detected in HCC tissues than in normal liver tissues and is closely associated with progression, recurrence, and the prognosis (113). Inhibitors of PD-1 and PD-L1 can block the interaction between these proteins and thus reverse T-cell activity for tumor killing. Following the clinical trials CheckMate-040 (9), KEYNOTE-224 (11), and IMbravel50 (114), the PD-1 inhibitors nivolumab and pembrolizumab and the PD-L1 inhibitor atezolizumab were approved for HCC treatment. In addition, other PD-1 inhibitors, such as tirelizumab and carrelizumab, and PD-L1 inhibitors, such as atirizumab, valileumab, and avelumab, have shown encouraging efficacy in clinical trials as HCC therapies (115).

Nivolumab (anti-PD-1) is a second-line ICI approved by the FDA in September 2017 for HCC patients who were treated with or intolerant to sorafenib. In a phase I/II clinical trial (CheckMate-040) with a cohort of 262 patients with advanced HCC, 48 patients in the dose-escalation cohort received 0.1–10 mg/kg nivolumab every 2 weeks for up to 2 years, while the other 214 patients in the dose-expansion cohort received 3 mg/kg nivolumab (9). The objective remission rates (ORRs) were 15% and 20% during the dose escalation and dose expansion periods, respectively. In the dose escalation cohort, the median OS was 15 months, and the median duration of efficacy was 17 months; in the dose expansion cohort, although the median OS could not be calculated, the median duration of efficacy was 9.9 months. This trial provides a rationale for the use of nivolumab as first-line therapy for patients with advanced HCC. A phase III trial (CheckMate-459) evaluated the efficacy of nivolumab versus sorafenib as a first-line treatment for patients with advanced HCC. The median OS was 16.4 months in the nivolumab group and 14.7 months in the sorafenib group (116).

Pembrolizumab (anti-PD-1) is a second-line ICI that was approved by the FDA in November 2018 for HCC patients who experienced treatment failure with sorafenib. A phase II clinical trial (KEYNOTE-224) with a cohort of 104 HCC patients reported an ORR of 17% (95% CI 11–26), median OS of 12.9 months (95% CI 9.7–15.5), and median progression-free survival (mPFS) of 4.9 months (11). In a randomized controlled phase III trial (KEYNOTErate-240), a median OS and PFS of 13.9 months and 3 months, respectively, were reported in the pembrolizumab group compared with 10.6 months and 2.8 months, respectively, in the placebo group. The ORR of the pembrolizumab group was 18.3%, which was significantly higher than the ORR of 4.4% for the placebo group (117).

Tislelizumab is a humanized antibody against PD-1 that was approved for HCC by the China National Medical Products Administration (NMPA) based on the results of the randomized phase II/III clinical trial RATIONALE-301 (NCT03412773). Although the OS of patients receiving the combination therapy was not superior to that of patients treated with sorafenib, the noninferiority median OS was 15.9 months for patients with combined therapy compared with 14.1 months for patients with sorafenib (*P* = 0.0398) (118). An international multicenter phase IA/IB clinical study (NCT02407990) indicated that tislelizumab monotherapy produced durable responses in patients with advanced tumors. The overall response rate (ORR) was 12.2%, and the disease control rate (DCR) was 51% in a cohort of 50 patients with HCC (119).

##### 4.1.1.3 Other ICIs

TIM-3 modulates the immune microenvironment of HCC by mediating T-cell depletion and apoptosis and enhancing Treg-mediated immune suppression (120). Preclinical studies have documented the antitumor efficacy of TIM-3 blockade alone or in combination with PD-1 inhibitors (121–123). Currently, a phase I clinical study of TSR-022, an anti-TIM-3 antibody, is ongoing in patients with advanced cancers, including HCC, as a first-in-man study (NCT02817633).

Although LAG-3 is not expressed on naive T cells, its expression can be induced on CD4+ and CD8+ T cells with sustained antigen stimulation (124, 125). LAG-3 blockade was shown to restore the cytotoxic activity of T cells for tumor killing (126, 127). Evidence suggests that LAG-3 signal transduction exerts a negative regulatory effect on Th1 cell proliferation and activation and cytokine secretion (128). In the tumor microenvironment, tumor cells use this pathway to avoid immune surveillance. Relatlimab was the first LAG-3 blocking antibody in clinical development (129). It was evaluated in clinical trials against solid tumors and hematologic malignancies as a monotherapy or in combination with nivolumab (anti-PD-1 mAb), and preliminary data showed that it was relatively well tolerated and exhibited clinical efficacy (130, 131).

TIGIT is expressed on activated T cells, NK cells, and B cells and inhibits immune cell-mediated antitumor responses (132, 133). TIGIT and the immunoactivating receptor CD226 trigger a signaling pathway similar to CD28/CTLA-4 to regulate tumor immune responses (134). In preclinical studies, anti-TIGIT antibodies combined with anti-PD-1/PD-L1 drugs had a synergistic effect (135, 136). According to related studies, TIGIT expression is elevated on lymphocytes infiltrating HCC tissues and may be involved in tumorigenesis and progression. At present, several exciting new drugs targeting TIGIT, such as tiragolumab, vibostolimab, etigilimab, ociperlimab, and domvanalimab, are in preclinical and clinical trials.

#### 4.1.2 Combination of ICIs

Based on the temporospatial expression patterns of CTLA-4 and PD-1/PD-L1, dual ICI therapy further improves therapeutic efficacy (15, 137). In the CheckMate-040 trial (Cohort 4), a combination of ipilimumab (anti-CTLA-4) and nivolumab (anti-PD-1) was administered to HCC patients with sorafenib treatment failure (15). The recommended regimen was ipilimumab (3 mg/kg) plus nivolumab (1 mg/kg) every 3 weeks for 4 cycles, followed by nivolumab (240 mg) biweekly, which yielded an ORR of 32% and a medium duration of response (DOR) of 17 months. This impressive result encouraged the approval of the ipilimumab plus nivolumab strategy after sorafenib by the FDA in March 2020 (137). In a phase I/II clinical trial of patients with advanced HCC who were previously treated with sorafenib, the regimen of tremelimumab (anti-CTLA4, 1500 mg) plus durvalumab (anti-PD-L1, 300 mg) every 4 weeks produced an ORR of 24% with a median OS of 18.7 months (17) (**Table 1**).

**Table 1:** Summary of clinical trials investigating ICIs combination therapy in HCC.

Clinical trail	N	Target	Treatment	Stage	Primary endpoint	Adverse event					Ref.
						Grade ≥3		Most common grade 3-4	Leading to discontinuation	Leading to death	
Monotherapy											
NCT01658878	262	PD-1	Nivolumab	I/II	ORR: 15% (n=48, dose escalation) vs. 20% (n=214, dose expansion)	Dose escalation (25%) vs. dose expansion (19%)		Lipase increase (13%); elevated serum AST (10%) vs. elevated serum AST (4%) and ALT (2%)	6% vs. 11%	0% vs. 0%	(9)
NCT02576509	743	PD-1	Nivolumab	III	OS: 16.4 months (n=371)	22%		Serum AST elevation (6%); pruritus (1%); diarrhoea (1%)	4%	1%	(10)
NCT02702414	104	PD-1	Pembrolizumab	II	ORR: 17.0%	26%		Elevated serum AST (7%) and/or ALT (4%); fatigue (4%)	23%	1%	(11)
NCT03389126	30	PD-L1	Avelumab	II	ORR: 10.0%	23%		Elevated serum AST/ALT (36.7%); neutropenia (3.3%); thrombocytopenia (3.3%)	7%	0%	(12)
NCT01008358	20	CTLA-4	Tremelimumab	II	PRR: 17.6%, DCR: 76.4% (n=17)	–		Elevated serum AST (45%) and/or ALT (25%); hyponatraemia (30%)	–	–	(13)
NCT02989922	217	PD-1	Camrelizumab	II	ORR: 14.7%, OSR: 74.4%, OS: 13.8 months	22%		Serum AST elevation (5%); hyperbilirubinaemia (3%); neutropenia (3%)	4%	1%	(14)
ICIs Combinations											
NCT01658878	148	PD-1 + CTLA-4	Nivolumab + Ipilimumab	I/II	ORR: 32% (n=50, arm A)/27% (n=49, arm B)/29% (n=49, arm C)	53% (arm A)		Elevated serum AST (16%); lipase (12%) and/or ALT (8%)	18%	2%	(15)
NCT03222076	30	PD-1 + CTLA-4	Nivolumab + Ipilimumab	II	TRAEs (grade ≥ 3): 43% (n=14, Nivolumab + Ipilimumab) vs. 29% (n=13, Nivolumab)	43%		Elevated serum AST (29%) and/or ALT (29%)	0%	0%	(16)
NCT02519348	332	PD-L1 + CTLA-4	Durvalumab + Tremelimumab	I/II	ORR: 24% (n=75, T300/D), PFS: 2.2 months, OS: 18.7 months	37.8%		Elevated serum AST (12%); lipase (7%) and/or amylase (7%)	10.8%	1%	(17)
ICI + Anti-angiogenes											
NCT03299946	15	PD-1 + TKIs	Nivolumab + Cabozantinib	I	TRAEs (grade ≥ 3): 13.3% (n=15)	13.3%		Fatigue (53.3%); nausea (33.3%)	40%	0%	(18)
NCT03755791	740	PD-L1 + TKIs	Atezolizumab + Cabozantinib	III	PFS: 6.8 (n=649, combination) vs. 4.2 (n=188, control) months, OS: 15.4 vs. 15.5 months (control)	57%		Elevated serum AST (9%) and/or ALT (8%), hypertension (9%), diarrhoea (4%)	14%	0%	(19)
NCT02715531	243	PD-L1 + VEGF	Atezolizumab + Bevacizumab	I	ORR: 36%(n=104), PFS: 5.6 (n=60) vs. 3.4 months (n=59, control)	20%		Hypertension (14%); proteinuria (7%); elevated serum AST (5%)	17%	3%	(20)
NCT03434379	501	PD-L1 + VEGF	Atezolizumab + Bevacizumab	III	OSR at 12 months: 67.2% (n=336) vs. 54.6% (n=165, control), PFS: 6.8 vs. 4.2 months (control)	56.5%		Hypertension (15.2%), elevated serum AST (7%) and/or ALT (3.6%)	15.5%	0%	(21)
NCT03006926	104	PD-1+ VEGFR	Pembrolizumab + Lenvatinib	Ib	ORR: 46% (mRECIST)/36% (RECIST v1.1), DOR: 8.6 months (mRECIST)/12.6 (RECIST v1.1) months	67%		Hypertension (17%); serum AST elevation (11%); diarrhoea (5%)	18%	3%	(22)
NCT03794440	595	PD-1 + VEGF	Sintilimab + IBI305	II/III	ORR: 20.5% (n=380) vs. 4.1% (n=191, control), PFS: 4.6 vs. 2.8 months	33.7%		Hypertension (14%); decreased platelet count (8%); proteinuria (5%)	13.7%	2%	(23)
ICI + Locoregional therapy											
NCT01853618	32	CTLA-4	Tremelimumab + RFA or CA or TACE	I	TRAEs: 13.0%	53%		Elevated serum AST (22%) and/or ALT (9%); hyperbilirubinemia (9%)	13%	0%	(24)
NCT03033446	39	PD-1	Nivolumab + Y-90 Radioembolization	II	ORR: 31% (n=36)	14%		Ascites (3%); fever (3%); liver abscess (3%)	6%	0%	(25)
ICI + Chemotherapy											
NCT03092895	157	PD-1+ VEGF	Camrelizumab + Apatinib or fluorouracil + calcium/folinate + oxoliplatin or gemcitabine + oxoliplatin	II	ORR: 26.5% (n=34), DCR: 79.4%	85.3%		Decreased neutrophil count (55.9%) and/or white blood cells (38.2%) and/or platelet count (17.6%)	0%	0%	(26)

PD-1, programmed cell death 1; PD-L1, programmed death ligand 1; CTLA-4, cytotoxic T-lymphocyte antigen 4; ALT, alanine

aminotransferase; AST, aspartate aminotransferase; TKIs, tyrosine kinase inhibitors; VEGF, vascular endothelial growth factor; VEGFR, vascular endothelial growth factor receptor; RFA, radiofrequency ablation; CA, chemoablation; TACE, transarterial chemoembolization; ORR, objective response rate; TRAEs, treatment-related adverse effects; DOR, duration of response; OS, overall survival; OSR, overall survival rate; PFS, progression-free survival; DCR, disease control response; RECIST v1.1, response evaluation criteria in solid tumors version 1.1; mRECIST, modified response evaluation criteria in solid tumors; –, not reported.

#### 4.1.3 ICIs combined with molecular targeted drugs

Molecular targeted drugs, such as sorafenib, regorafenib, and lenvatinib, promote tumor antigen presentation, thus activating T cells to improve the efficacy of immunotherapy (138). VEGF is a chemokine that induces angiogenesis and contributes to tumor invasion and metastasis. In addition, it helps recruit and induce the activation of Tregs, TAMs, and MDSCs to shape an immunosuppressive microenvironment (139). Although anti-VEGF therapy alone reduces the activity of Tregs and MDSCs and induces cytotoxic T-cell infiltration, it shows only modest efficacy against HCC (140, 141).

Based on a phase Ib clinical trial of patients with unresectable HCC, the PD-L1 inhibitor atezolizumab plus the VEGF inhibitor bevacizumab significantly suppressed HCC progression and improved PFS compared to atezolizumab monotherapy (20). In 2020, a randomized phase III clinical trial, IMbrave150 (NCT03434379), was approved by the FDA to compare the efficacy of atezolizumab plus bevacizumab (n = 336) with sorafenib (n = 165) in 501 treatment-naive patients with unresectable Child–Pugh A HCC (21, 142). The atezolizumab plus bevacizumab regimen mediated dual immune modulation by inhibiting PD-L1 and VEGF signaling and showed an OS benefit compared with sorafenib. The median survival time (MST) was 19.2 months (HR 0.66; 95% CI 17.0–23.7) in the atezolizumab plus bevacizumab group and 13.4 months in the sorafenib group. Currently, the atezolizumab plus bevacizumab combination is the standard regimen for patients with advanced Child–Pugh A HCC.

ORIENT-32 (NCT03794440) is a phase II/III clinical trial comparing the PD-1 inhibitor sintilimab plus a bevacizumab biosimilar, IBI305, with sorafenib in Chinese patients with unresectable or metastatic HCC (23). Sintilimab plus IBI305 produced both PFS and OS benefits compared with sorafenib, and had an acceptable safety profile. Another PD-1 inhibitor, camrelizumab, plus a tyrosine kinase inhibitor (TKI), apatinib, was reported to effectively prolong the survival of patients with mid- and advanced-stage HCC with good tolerance (143).

Lenvatinib is a multi-TKI that targets FGFRs, VEGFRs, PDGFRα, and KIT that was approved by the FDA in August 2018 as the first-line drug for patients with unresectable HCC (144). The combination of pembrolizumab and lenvatinib was evaluated in a phase Ib trial of untreated HCC patients, generating an ORR of 46%, a median OS of 22 months, and a median PFS of 9.3 months (22). Although grade ≥ 3 treatment-related adverse events (TRAEs) occurred in up to 67% of the patients, these toxicities were controlled by dose modifications and interruptions (22).

#### 4.1.4 ICIs combined with chemotherapy

The combination of chemotherapy and immunotherapy produces multiple complex effects. While killing cancer cells, chemotherapy stimulates the release of antigens from tumor cells, reduces the number of immunomodulatory cells, and induces an increase in the number of antitumor effector T cells (145–147). A phase II study (NCT03434379) evaluated camrelizumab plus the FOLFOX4 or GEMOX regimens as a first-line strategy for patients with advanced primary HCC or cholangiocarcinoma (26). The ORR was 26.5%, and the disease control rate (DCR) was 79.4% in 34 patients. In terms of safety, 85.5% of HCC patients experienced TRAEs of grade ≥ 3, with myelosuppression and allergic reactions reported as the most common TRAEs.

#### 4.1.5 ICIs combined with radiotherapy

Radiation therapy (RT) induces tumor cell apoptosis and the release of tumor-specific antigens to activate the immune system, suggesting that radiotherapy may exert a synergistic effect on tumor killing with immunotherapy. Numerous preclinical studies have shown that the combination of RT and ICIs produces synergistic effects and significant tumor suppression (148–151). A study reported results from five patients with large unresectable HCC who were treated with nivolumab after SBRT. They all achieved an ORR of 100%, with two patients in complete remission and three patients in partial remission (152). Another combination of Y-90-RE with nivolumab resulted in an ORR of 31%, and the combination proved to be safe and tolerable, with only a minority (11%) of treated patients with advanced HCC experiencing grade 3 or higher TRAEs (153).

#### 4.1.6 ICIs combined with TACE

TACE is one of the main treatments for patients with inoperable HCC. It causes ischemic necrosis of the tumor tissue by embolizing the artery with chemotherapeutic agents delivered to the hepatic artery. TACE therapy for patients with unresectable HCC has been proven effective, but the long-term survival of patients is not satisfactory. In a clinical trial evaluating the safety of TACE plus pembrolizumab for HCC, TACE plus pembrolizumab had a tolerable safety profile with no synergistic toxicity (154).

#### 4.1.7 ICIs combined with RFA

RFA is the first-line therapy for early local ablation and plays a leading role in the treatment of HCC. A large body of evidence suggests that ablative therapy not only physically eliminates tumors but also activates systemic antitumor immune responses and suppresses immunosuppressive effects (155–157). In a clinical study evaluating the efficacy of tremelimumab plus RFA for advanced HCC, it showed that 5 of the 19 evaluable patients achieved partial remission, 12 of 14 HCV+ patients had a significantly reduced viral load, and patients who experienced a clinical benefit had significantly increased CD8+ T-cell numbers, with a median OS of 12.3 months and median time to tumor progression of 7.4 months (24).

#### 4.1.8 Immune-related adverse events

Although ICIs activate antitumor immunity, they inevitably alter the original immune homeostasis, resulting in a hyperactive immune response and a series of adverse reactions similar to autoimmune diseases. irAEs involve multiple organs but mainly affect the skin, liver, lung, gastrointestinal tract and endocrine organs (158). HCC usually develops from cirrhosis and has systemic manifestations (159). Therefore, the symptoms caused by multiple organs overlap with irAEs and increase their severity. Overall, the PD1 inhibitors pembrolizumab and nivolumab result in severe irAEs in 10-20% of patients with advanced HCC and over 30% of patients with other cancer types (160). However, the incidence of liver-related irAEs is higher in patients with HCC than in patients with other tumor types. Anti-PD1 monotherapy induces hepatitis-related enzymes in 14% of HCC patients but in only 3% of patients with other cancer types (161, 162).

Principally, irAEs are attributed to the hyperactive immune system. The role of the CTLA-4 and PD-1 checkpoints in autoimmunity and immune homeostasis is fundamental to our understanding of the mechanisms of irAEs. Although the precise mechanism of irAEs is not fully understood, the incidence of irAEs involves complex dynamic changes, including the regulation of effector T-cell and Treg activity, toxic effects of macrophages and granulocytes, cytokine release, and antibody production by B cells (160). The life-threatening nature of irAEs limits the application of ICIs. Therefore, biomarkers predicting the severity of irAEs are key points to improve patient survival and quality of life. The possible biomarkers for irAEs are summarized below. (1) Autoimmune diseases are caused by hyperactive or suppressive immune-related cells, and the immunosuppressive microenvironment is a characteristic of tumor initiation and progression. The investigation of the heterogeneity of immune-related cells may promote a better understanding of immune disorders, thus improving the prediction and management of irAEs. (2) Cytokines, such as interleukin, TNF-α, and IFN-γ, are indispensable for cancer immunotherapy. Their secretion changes remarkably with ICI treatment and might be involved in irAEs. Previous studies examining immunotherapy revealed that the levels of cytokines are closely correlated with the incidence of irAEs, suggesting the possibility that cytokines may serve as biomarkers for irAEs. (3) Tumorigenesis is acknowledged as a consequence of genetic mutations, which may interfere with communication between immune cells and tumor cells. NGS and single-cell RNA sequencing technology may enable the extensive screening of genetic mutations as potential biomarkers for irAEs. (4) Serum biomarkers reflecting the tumor burden were revealed to be associated with the response to ICIs. Therefore, studies investigating the prediction of irAEs are worthwhile.

### 4.2 Adoptive cell therapy

Adoptive cell therapy (ACT) is a host cell-based immunotherapy that endows lymphocytes with long-lasting antitumor immunity and produces low cytotoxic effects on normal cells. The basic principle involves isolating host lymphocytes, followed by their enrichment, activation, and amplification *in vitro via* stimulation with cytokines or peptides or *via* genetic modification. The modified cells are then infused into the host to exert cytotoxic effects on autologous tumor cells (163). The most commonly used ACT-based therapies for HCC include chimeric antigen receptor (CAR)-modified T cells, cytokine-induced killing (CIK) cells, and T-cell receptor (TCR)-engineered T cells (**Table 2**).

**Table 2:** Summary of clinical trials investigating ACT in HCC.

Clinical trail	N	Target	Treatment	Stage	Primary endpoint	Adverse event				Ref.
						Grade 1-2	Most common grade 1-2	Grade≥3	Most common grade 3-4	
NCT02395250	13	GPC3	CAR-T cells	I	ORR: 15.4%, OSR: 10.5% (3-year), 42.0% (1-year), and 50.3% (6 months)	85%	Pyrexia (85%); CRS (62%); CRP increased (54%); chills (54%); cough (54%)	92%	Decreased lymphocyte count (92%)	(27)
NCT03980288	6	GPC3	CAR-T cells	I	ORR: 16.7%, DCR: 50%, PFS: 4.2 months	50%	–	50%	Lymphocyte depletion (50%)	(28)
NCT00769106	200	–	CIK cells	III	TTR: 13.6 (n=100) vs. 7.8 months (n=100, control)	11.8%	Fever (7.8%); abdominal pain (5.8%)	0%	0%	(29)
NCT00699816	230	–	CIK cells	III	RFS: 44.0 (n=115) vs. 30.0 months (n=115, control)	35%	Fever (9%); chills (8%)	0%	0%	(30)
–	132	–	CIK cells	II	OSR: 74.2% (3-year), 53.0% (2-year), and 50.3% (1-year) (n=66)	41%	Leukopenia (41%); fever (29%); headache pain (12%)	0%	0%	(31)
NCT03899415	8	HBV	TCR-T cells	I	TRAEs: 25.0%, TTP: 6.18 months, OS: 33.1 months (n=8)	12.5%	Elevated serum ALT (12.5%)	37.5%	Elevated serum AST (12.5%) and/or ALT (12.5%) and/or GGT (12.5%)	(32)
NCT02719782	10	HBV	TCR-T cells	I	TTP: 1.3 months, OS: 14 months (n=4)	67%	Fever (67%)	0%	0%	(33)

ACT, adoptive cell therapy; GPC3, glypican 3; CAR-T, chimeric antigen receptor T cells; ALT, alanine

aminotransferase; AST, aspartate aminotransferase; CRS: cytokine release syndrome; CRP, C-reactive protein; GGT, γ- glutamyltransferase; ORR, objective response rate; OSR, overall survival rate; DCR, disease control response; PFS, progression-free survival; CIK, cytokine-induced killer cells; TTR, time to recurrence; RFS, recurrence-free survival; HBV, hepatitis B virus; TCR-T, T cell receptor engineered T cells; TRAEs, treatment-related adverse effects; TTP, time to progression; OS, overall survival; –, not applicable..

#### 4.2.1 CAR-T cells

CAR is a fusion protein composed of a tumor-associated antigen (TAA) binding domain, an extracellular hinge region, a transmembrane domain, and an intracellular signal domain (164, 165). Following CAR modifications, T lymphocytes recognize a broader range of target antigens than the natural TCR (164) and participate in the identification of cancer cells independently of the major histocompatibility complex (MHC). Glypican-3 (GPC3) is specifically expressed on the surface of HCC cells and was used as the TAA for CAR-T cell construction (166, 167). CAR-T cells targeting GPC3 have proven effective *in vitro* and in animal models, with a clinically significant prolongation of survival (168). In recent phase I trials (NCT02395250 and NCT03146234) comprising 13 patients with advanced HCC receiving CAR-T-cell infusions, two patients showed partial responses, and one patient showed persistent stable disease (SD) (27). In another ongoing phase I clinical trial (NCT03198546), complete tumor disappearance was observed in patients with GPC3+ advanced HCC after 30 days of treatment with intratumor injections of 19.9 × 10^8^ CAR-GPC3 T cells (169).

Although CAR-T cell therapy has made breakthroughs in the treatment of hematologic malignancies through its targeting specificity, CAR-T cell therapy for solid tumors, including HCC, still faces many challenges. These challenges are mainly attributed to the intricate immune microenvironment within solid tumors, with specific histopathological features leading to hypoxia, a low pH, immunosuppressive cells, an increased number of inhibitory checkpoints, and higher levels tumor-derived cytokines (170, 171). These characteristics make T-cell infiltration in local tumor tissues more challenging (172), thus affecting the cytotoxic antitumor effect of T cells. A number of studies have been conducted to overcome the detrimental effects of the tumor microenvironment in solid tumors, including the modification of CAR-T cells by knocking down PD-1 expression and the use of CAR-T cells in combination with ICIs (173–175).

#### 4.2.2 CIK cells

CIK cells are heterogeneous and non-MHC-restricted lymphocytes consisting of T cells (CD3^+^CD56^−^), NK cells (CD3^−^CD56^+^), and NKT cells (CD3^+^CD56^+^) (176). CD3^+^CD56^+^ NKT cells are the main effector cells exerting antitumor activity due to the higher proportion of CD8^+^ cells, more differentiated effector cells, and higher granzyme A content (177). CIK cells are produced by the expansion and activation of peripheral blood mononuclear cells (PBMCs) in response to stimulators such as an anti-CD3 antibody, IFN-γ, and IL-2 (178). With the advantages of non-MHC-restricted cytotoxicity, dual function of T cells and NK cells, high proliferation rate, high specificity for tumors, and low impact on normal cells (179), CIK cells are applied to both hematological malignancies and solid tumors.

A randomized phase II trial of CIK cells for HCC revealed that CIK cell therapy significantly prolonged OS and recurrence-free survival (RFS) compared with the control group (31). In a multicenter phase III clinical trial consisting of 230 HCC patients, adjuvant immunotherapy with activated CIK cells was administered to HCC patients that treated with surgical resection, RFA, or a percutaneous ethanol injection. The median PFS was 44 months in the CIK group and 30 months in the control group. The CIK group showed lower HRs for all-cause death (0.21; 95% CI 0.06–0.75; *P* = 0.008) and cancer-related death (0.19; 95% CI 0.04–0.87; *P* = 0.02). Although the overall frequency of adverse events (AEs) was higher in the CIK group than in the control group (62% vs. 41%; *P* = 0.002), the severe AE incidence did not differ significantly (7.8% vs. 3.5%; *P* = 0.15) (30). A 5-year follow-up study (NCT01890291) revealed the sustained therapeutic efficacy of autologous CIK cell immunotherapy against HCC. The study enrolled 162 patients, with 89 in the CIK group and 73 in the control group. After an average follow-up of 68.5 months, the recurrence-free survival (RFS) rate in the CIK group was 44.8% compared to 33.1% in the control group. Furthermore, the CIK group showed a significant reduction in the risk of all-cause death with a hazard ratio of 0.33 (95% CI: 0.15-0.76, P = 0.006) (180). To date, several clinical trials have evaluated the role of CIK cells in the treatment of HCC and have obtained encouraging results, suggesting that CIK cells are a very promising immunotherapy for the treatment of HCC (29, 181, 182).

#### 4.2.3 TCR-T cell

TCR-T cell therapy is performed by extracting the α and β chain genes encoding the TCR from tumor antigen-induced effector T cells, introducing them into mature T cells using genetic engineering techniques, and then infusing the cells back into patients who lack tumor antigen-specific responsive T cells. TCR-T cells perform antigen recognition in an MHC-dependent manner (183) and have a wider range of applications than CAR-T cells. Hepatitis B virus (HBV) infection is the leading cause of HCC in Asia, accounting for 80% of cases (184). HBV infection stimulates the production of high-affinity T-cell receptors (TCRs), which may provide a potential therapeutic strategy for targeting HBV-infected cells. However, a major concern is that viral antigens may also be expressed on nonmalignant liver tissue in patients with HBV-associated HCC, posing a risk of severe liver injury (185). Current candidate TAAs for HCC TCR-T therapy include New York esophageal squamous cell carcinoma 1 (NY-ESO-1) (186), alpha-fetoprotein (AFP) (187), GPC3 (188), and human telomerase reverse transcriptase (hTERT) (189). NY-ESO-1 is the most commonly used target antigen in clinical trials of TCR-T cells. NY-ESO-1 is expressed in approximately 45% of HCC tumors (190); therefore, NY-ESO-1-specific TCR-T cells have been used in clinical therapeutic trials for HCC (NCT01967823, NCT02869217, and NCT03159585). Recently, Docta et al. obtained an HLA-A2+/AFP-specific TCR (191), and a clinical trial of this TCR-T cell population for HCC is underway to confirm its effectiveness in treating HCC by examining AFP expression and T-cell infiltration in biopsied tissues (NCT03132792). In contrast to AFP, fewer studies have been conducted on GPC3-specific TCRs. A study obtained HLA-A2/GPC3367-specific TCRs by transfecting HLA-A2 with GPC3 into HLA-A2-negative donor DCs and coculturing them with host T cells; the expression of this receptor on T cells enabled them to recognize and kill GPC3-positive HCC cells (188).

### 4.3 Cytokine therapy

Cytokines are an important component of the immune system and promising therapeutic targets in liver diseases due to their importance in modulating immune and inflammatory responses (192). In recent years, many cytokines, such as IL-2, IL-15, IL-21, GM-CSF, and IFN-α, have proven effective in preclinical tumor models and are used as potential options for cancer immunotherapy (193). With the ability to mediate antiviral, antitumor, and other immune responses, IFN-α reduces the mortality and early relapse rates of HCC patients after curative treatment (194). In addition, adjuvant IFN therapeutic efficacy on postoperative recurrence differed between patients with HBV-related HCC and patients with HCV-related HCC; therefore, the adjuvant IFN strategy should be used according to the hepatitis background (195). A preclinical study suggested that hepatocyte growth factor (HGF) may be involved in the progression of HCC (196). Two studies have documented that pretreatment serum HGF levels are potential independent predictors of OS in prospective cohorts of HCC patients (197, 198). Notably, lower HGF levels at the initiation of treatment are often associated with longer OS and PFS benefits from sorafenib treatment (198). IL-2 is capable of promoting T-cell proliferation and activation, which is essential for tumor-killing activity. In a clinical trial of patients with inoperable HCC, IL-2 administration prolonged OS (199). A study of patients with HCC treated with sorafenib therapy evaluated the prognostic value of pretreatment serum IL-6 levels. In both the discovery and validation cohorts, higher pretreatment serum IL-6 levels (threshold: 4.28 pg/mL) were an independent predictor of shorter OS. However, this result was unrelated to the efficacy of sorafenib, as PFS and TTP were similar, regardless of pretreatment IL-6 levels. Additionally, pretreatment IL-6 levels were not associated with macrovascular invasion or extrahepatic metastasis (200). A recent study using cellular models showed that inhibition of IL-6-related pathways may reduce resistance to sorafenib (201). TGF-β serves as a tumor suppressor at early stages of tumorigenesis. However, with tumor progression, TGF-β loses its growth-inhibitory ability and initiates the EMT and cell migration (202). Galunisertib (LY2157299), a novel TGF-β inhibitor, was evaluated in a phase II trial of HCC patients with disease progression on sorafenib therapy. The median OS for the cohort with AFP levels < 200 ng/mL was 17 months compared to 8.4 months for patients with AFP levels > 200 ng/mL. Notably, an obvious improvement in OS was noted in patients with TGF-β1 levels that were reduced by more than 20% (203, 204). In addition, preclinical studies have shown that galunisertib modulates the inhibition of T-cell proliferation and shows potential synergy with PD-1/PD-L1 inhibitors (205). Galunisertib is currently being evaluated for efficacy and safety in the treatment of HCC patients in several ongoing clinical trials (NCT02240433, NCT01246986, NCT02178358, NCT02906397, and NCT02423343). However, only high concentrations of cytokines are able to reach the tumor microenvironment, and parenterally administered cytokines have difficulty reaching effective concentrations and exerting their effects on the tumor microenvironment. The combination of cytokines with other immunotherapies is currently being clinically investigated to enhance their antitumor capacity and avoid these obstacles.

### 4.4 Therapeutic vaccine

Therapeutic vaccines are active immunotherapies in which TAAs (including peptides, tumor cells, and viruses) are introduced into patients to overcome the immunosuppressive tumor microenvironment and further activate the patient’s immune system to produce a tumor-specific response with enhanced potency (206). The identification of target TAAs is the most critical step in exerting a specific antitumor response. Several HCC peptide vaccines targeting TAAs, such as GPC3, AFP, and hTERT, have been developed for use as immunotherapeutic targets for vaccines (207).

The GPC3 peptide is considered an ideal vaccine for HCC treatment due to its overexpression on HCC tumor cells and low level on normal cells (208). A phase I clinical study of patients with advanced HCC showed that the GPC3 vaccine was well tolerated and induced a GPC3-specific CTL response in 30/33 patients (91%) (209). In a phase II study of 41 HCC patients who underwent surgical resection, adjuvant GPC3 vaccine administration significantly reduced the recurrence rate (1- and 2-year recurrence rates of 24% vs. 48% and 52.4% vs. 61.9%, respectively; *P* = 0.047, 0.387). In addition, the postoperative administration of the GPC3 peptide vaccine was shown to prolong RFS (210).

AFP is a classic diagnostic marker for HCC due to its specific expression on HCC cells (211). AFP blockade reduces the proliferation and promotes apoptosis of liver cancer cells (212). In a clinical trial, two AFP-positive HCC patients received an AFP vaccine, which showed good tolerance and safety without clinically significant adverse events (213).

### 4.5 Oncolytic viruses

Oncolytic viruses (OVs) are a group of genetically modified viruses that have acquired the ability to kill tumor cells in a targeted manner without damaging normal cells. During the killing of tumor cells by oncolytic viruses, the antitumor immune response is activated in the body, and the inflammatory response generated by viral infection further promotes the immune response (214). OVs are usually divided into two categories, namely, natural viruses (including Newcastle disease virus and eutherian virus) and genetically modified viruses (including adenovirus and herpes simplex virus) (215). JX-594 is an engineered oncolytic virus that selectively targets tumors by inactivating viral thymidine kinase (vTK) (216). In a phase II clinical trial of the cowpox vaccine JX-594 for the treatment of HCC, JX-594 was well tolerated even at high doses. The study showed a positive correlation between the dose of JX-594 and survival benefit, with a longer median OS for the high-dose group than the low-dose group (14.1 months and 6.7 months for the high- and low-dose groups, respectively) (216). Currently, two clinical studies of OVs in combination with anti-PD-1 antibodies for the treatment of HCC (NCT04612504 and NCT05061537) are ongoing. A recent study showed that treatment with the cancer-favoring vaccinia virus (CVV) resulted in a significantly lower incidence of metastasis than treatment with sorafenib alone in an animal model (217). Although OVs for HCC show promising clinical applications, more clinical trials are needed to further validate their efficacy and safety.

## 5 Conclusions

The tumor microenvironment and cancer immunotherapy have been at the forefront of cancer research in recent decades. Immune cells in the immune microenvironment of HCC tumors provide new targets for the next generation of immunotherapy. Immune checkpoint therapies targeting CTLA-4 and PD-1/PD-L1 are the main drugs used in the treatment of HCC, and other immunotherapies are rapidly evolving, including CAR-T cells, TCR-T cells, therapeutic vaccines, and OVs. Although response rates to ICIs in HCC patients are modest, more new treatment modalities will emerge as our understanding of the interaction of ICIs with adaptive and innate immune responses improves. Many strategies have been developed that combine ICIs with other first-line therapies (including chemotherapy, local therapies, and targeted therapies) in ideal combinations, and these new therapies have provided palliation and prolonged the survival of patients with HCC.

## Author contributions

MZ and HH wrote the manuscript. FH and XF revised and edited the manuscript. All authors read and approved the final version of the manuscript.

## Glossary

**Table:**

AFP	alpha-fetoprotein
ACT	Adoptive Cell Therapy
α-SMC	α-smooth muscle cell
CAR	chimeric antigen receptor
CCL26	C-C motif chemokine 26
CIK	cytokine-induced killing
CTLA-4	cytotoxic T lymphocyte associated protein 4
CAF	cancer-associated fibroblasts
CTL	cytotoxic T lymphocyte
CSC	sequester circulating cancer cells
CCA	cholangiocarcinoma
CXCL	chemokine (C-X-C motif) ligand
CYLD	cylindromatosis
CVV	cancer-favoring vaccinia virus
cDCs	conventional DCs
DC	dendritic cells
DCR	disease control rate
ECM	extracellular matrix
EMT	epithelial–mesenchymal transition
FDA	US Food and Drug Administration
FoxP3	forkhead box protein 3
FGF	fibroblast growth factor
FAP	fibroblast activating protein
GPC3	Glypican 3
GM-CSF	granulocyte-macrophage colony stimulating factor
HCC	Hepatocellular carcinoma
HSC	hepatic stellate cell
hTERT	telomerase reverse transcriptase
ICB	immune checkpoint blockade
ICIs	immune checkpoint inhibitors
IDO	indoleamine-2,3-dioxygenase
IFN-γ	interferon gamma
KCs	Kupffer cells
KIT	c-kit receptor
LPS	lipopolysaccharide
LSAMP	limbic system-associated membrane protein
LAG-3	lymphocyte-activation gene 3
MDSC	myeloid-derived suppressor cells
MC	mast cells
MST	median survival time
MFB	myofibroblast
MHC	major histocompatibility complex
NK	natural killer cells
NET	neutrophil extracellular traps
NKG2D	natural killer group 2D
NKG2A	natural killer group 2A
NGF	nerve growth factor
NY-ESO-1	New York esophageal squamous cell carcinoma 1
NMPA	national medical products administration
OS	overall survival
OVs	oncolytic viruses
ORR	objective remission rates
OS	overall survival
PD1	programmed cell death 1
PD-L1	programmed death ligand 1
PFS	progression-free survival
PBMC	peripheral blood mononuclear cells
PDGF	platelet-derived growth factor
PDGFR	platelet-derived growth factor receptor
pDCs	plasma cell-like DCs
RFA	radiofrequency ablation
RFS	recurrence-free survival
SIRT	selective internal radiation therapy
SBRT	stereotactic body radiation therapy
TAA	tumor-associated antigen
TACE	transarterial chemoembolization
TIL	tumor infiltrating lymphocytes
TAM	tumor-associated macrophages
TAN	tumor-associated neutrophils
TGF-β	transforming growth factor-β
TNF-α	tumor necrosis factor-α
TCR	T cell receptor
TIGIT	T cell immune receptor with Ig and ITIM domains
Tim-3	T cell immunoglobulin and mucin domain-containing protein 3
VEGF	vascular endothelial growth factor
VEGFR	vascular endothelial growth factor receptor.
